# Cross-Cultural Adaptation and Validation of The Nepali Version of The Prosthetic Limb Users Survey of Mobility Short-Form (Plus-M™/Nepali-12Sf) In Lower Limb Prosthesis Users

**DOI:** 10.33137/cpoj.v6i1.41310

**Published:** 2023-08-23

**Authors:** AR Bajracharya, S Seng-iad, K Sasaki, G Guerra

**Affiliations:** 1 Sirindhorn School of Prosthetics and Orthotics, Faculty of Medicine Siriraj Hospital, Mahidol University, Bangkok, 10700, Thailand.; 2 Department of Exercise and Sport Science, St. Mary's University, San Antonio, Texas, 78210, USA.

**Keywords:** Prosthetics, PLUS-M, 2MWT, Outcome Measurement, Survey, Mobility, Nepali, Lower Limb Prosthesis

## Abstract

**BACKGROUND:**

Objective mobility measurement of Nepali prosthesis users is lacking.

**OBJECTIVE:**

The objective of this study was to cross-culturally adapt, translate and evaluate construct validity of the Prosthetic Limb Users Survey of Mobility (PLUS-M™/Nepali-12 Short Form (SF)) instrument in lower limb prosthesis users residing in Nepal.

**METHODOLOGY:**

Two forward translations, review and reconciliation, back translation, expert review, developer review to create the PLUS-M™/Nepali-12SF. Psychometric testing for internal consistency, test-retest reliability and construct validity against the Two-Minute Walk Test (2MWT) and Amputee Mobility Predictor with Prosthesis (AMPPRO) were performed on sixty-six lower limb prosthesis users.

**FINDINGS:**

The majority of populations were with transtibial amputation 45 (68%), with transfemoral amputation 15 (23%), with knee disarticulation 5 (7.5%) and with syme's amputation 1 (1.5%). The most common cause of amputation among the population was trauma and the least was tumor. Chronbach's alpha for the PLUS-M™/Nepali-12SF was 0.90, mean T-Score was 52.90, test-retest intraclass correlation coefficient (ICC) was 0.94 (95% confidence interval 0.90–0.96). Construct validity with the 2MWT was good (r = 0.62, p< 0.001) and moderately positive with the AMPPRO (r = 0.57, p< 0.001).

**CONCLUSION:**

Our research evidenced that the PLUS-M™/Nepali-12SF had excellent reproducibility. The significance of this work is that it may allow for the measurement of mobility in austere locations of Nepal.

## INTRODUCTION

Recent data from the WHO-UNICEF Global Report on Assistive Technology (GReAT) estimates that approximately 2.5 million people are in need of assistive technologies now, and by 2050 that number will rise to 3.5 million people.^1^ Moreover, for persons residing in resource limited settings, only 3% may have access to vital assistive technologies. Several key recommendations have been provided in the GReAT report, one of which is to ensure effectiveness of assistive technology (AT) and actively involve AT users in this process. Lower limb prosthetics are an essential AT for persons with limb loss disabilities. The effectiveness of rehabilitation treatment must be assessed using reliable performance based and patient reported outcome measures.^2^ A plethora of instruments have been developed to better understand the effect of prosthetic treatment on mobility.^3–5^ The Prosthetic Limb Users Survey of Mobility (PLUS-M™) is a recently developed instrument for measuring lower limb prosthesis user mobility.^6^ This instrument was developed following rigorous patient reported outcome measurement procedures.^7^ The PLUS-M™ has good construct validity with Amputee Mobility Predictor (AMP)^4^ and Timed Up and Go test (TUG).^8^ The AMP provides clinicians a tool for determining prosthesis user activity level. The Two-Minute Walk Test (2MWT) also offers indices of prosthesis user walking capacity in a short and simple to administer assessment.^9^ These two assessments combined offer a practical means for gauging mobility in lower limb prosthesis users.^10,11^ In the event that a performance based mobility measurement cannot take place, the PLUS-M™ may serve well. Correlation between PLUS-M™ and mobility measures has previously indicated convergent construct validity.^6^

Understanding how well a prosthesis user can walk over the varied and sloped terrain in Nepal is important for the Nepali prosthetists. Nepali prosthesis users must ambulate in these harsh terrains for work, activities of daily living and leisure.^12^ Although the PLUS-M™ is available in many languages,^13,14^ it has yet to be translated and culturally adapted to Nepali. The objective of this study was to cross-culturally adapt, translate and evaluate construct validity of the PLUS-M™/Nepali-12 Short Form (SF) instrument in lower limb prosthesis users residing in Nepal.

## METHODOLOGY

### Setting

This study was approved by *Siriraj Institutional Review Board* and also by *Nepal Health Research Council*. All participants provided written informed consent prior to data collection. Sixty-six lower limb prosthetic user's age ≥18 who were independent and had received their prosthesis for at least six months were purposively selected from regional centers. These users had no neurological, musculoskeletal or pathologies which would have affected study participation. Participants unable to understand Nepali; users needing assistance of helper to walk, cognition problem, users with underlying medical conditions affecting mobility and <18 years of age were excluded from this study.

### Cross-cultural translation

Permission to proceed with translation was received from the instrument developer and a formal translation method was performed. The developers provided pertinent scoring and definition guides for instrument items. Two bilingual Nepal and English persons (physical therapist, prosthetist) independently established a Nepali version. Next, bilingual experts (medical doctor, physical therapist) reviewed possible discrepancies. Thereafter, a reconciled version was created and back translated by an American who speaks Nepali. This backward translation was sent to the developer; Professor Brian Hafner to review and incorporate or change if needed. The comments and suggestions from developer were incorporated in Nepali version by translator and revised back translation was again sent to the developer for additional feedback. The comments and suggestion were collected and incorporated in revised back translation and this version was provided to three independent bilingual experts (medical doctor, physical and occupational therapist). This revised addition was modified for precision before being sent to the developer for a final review.

The final PLUS-M™/Nepali 44 - items bank was pre-tested by cognitive interview with 7 participants with lower limb amputation, which helped to detect respondent interpretation. Concurrent probing took place in an interview by comparing each item with the help of a manual of definitions of terms and intentions of each question item provided by the developer.

### Psychometric evaluation

Demographic data, amputation date, level and etiology were recorded. The data collector evaluated performance-based outcome measures Two-Minute Walk Test (2MWT)^15^ and Amputee Mobility Predictor with Prosthesis (AMPPRO), and PLUS-M™ Nepali 12 item short form (PLUS-M™/Nepali-12SF). The participants were given the option of performing the PLUS-M™ Nepali-12SF first, followed by the 2MWT, and AMPPRO, or the 2MWT, and AMPPRO followed by the PLUS-M™/Nepali-12SF. The 2MWT and AMPPRO served as a basis for construct validity testing. In the 2MWT participants were instructed to walk as fast as possible without running along a flat rectangular outdoor walkway of 20 meter (65.6 ft.), and distance was recorded in meters. To explore test-retest reliability participants performed the PLUS-M™/Nepali-12SF once more two weeks later.

### Data analysis

SPSS v16 (IBM, Armonk, New York, USA) was used to analyze data. Characteristics of the participants were analyzed using a Pearson Chi Square test (p<0.05) and 95% confidence interval. Internal consistency was assessed via Chronbach's alpha, with ≥ 0.07 considered good internal consistency.^16^ An intra-class correlation coefficient (ICC) was employed to evaluate test-retest reliability. Construct validity was evaluated using a Pearson Correlation Coefficient, with r ≥ 0.6 = good to excellent and r < 0.6 = poor to moderate correlation.^17^

## RESULTS

**Figure 1:**
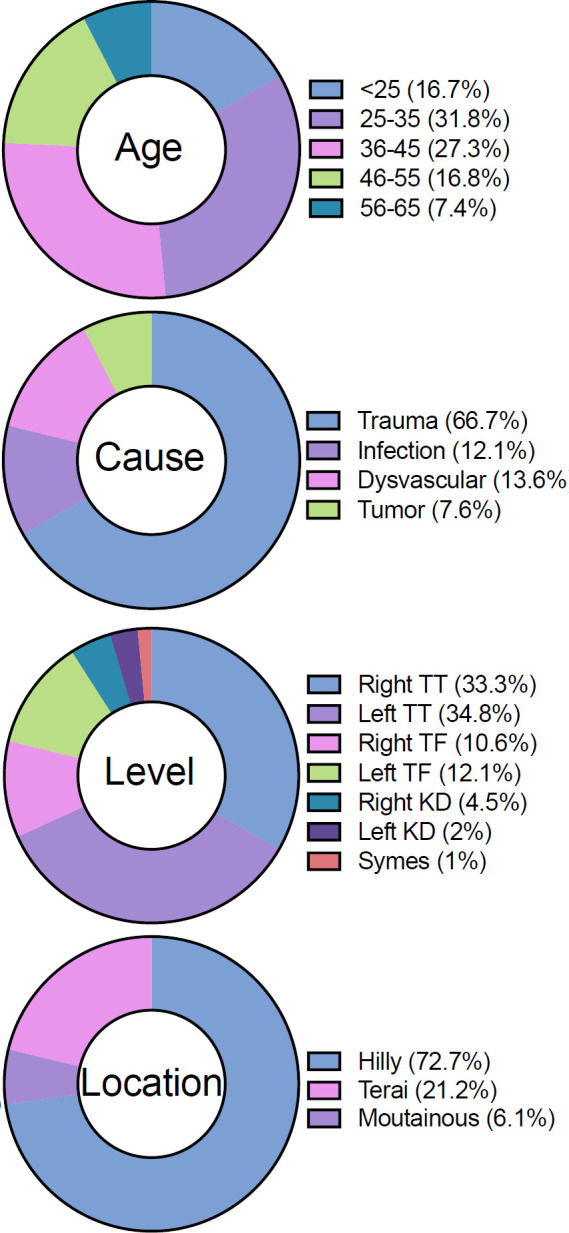
Demographics of study participants (n=66).

Sixty-six prosthesis users completed the study (37.26 ± 11.81 years old) (Figure 1).

**Figure 2:**
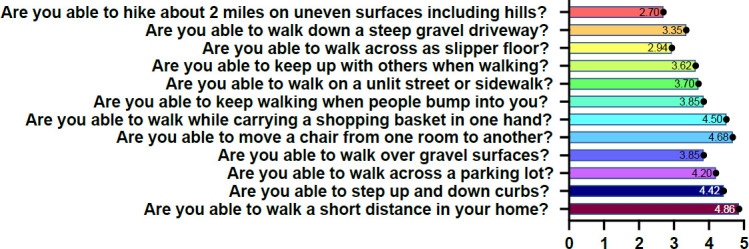
Mean participant response on PLUS-M™/Nepali-12SF. Note: Figure illustrates mean response on items of the instrument. Lower number indicates more difficulty performing the task. Solid large circle is mean, smaller circles are individual data points. Solid lines connect tests and rain cloud plots on the right indicate data distribution.

Figure 2 provided item and mean responses to the instrument. The overall Chronbach's alpha for the PLUS-M™/Nepali-12SF was 0.90 showing good internal consistency. This instrument scores using a T-Score which is a standardized score with a mean of 50 and standard deviation of 10. A higher T-Score is equal to a higher mobility and vice-versa. PLUS-M™/Nepali-12SF initial mean T-Score was 52.90, with standard deviation 5.6. The minimum score was 36.4 and maximum was 67.1.

**Figure 3:**
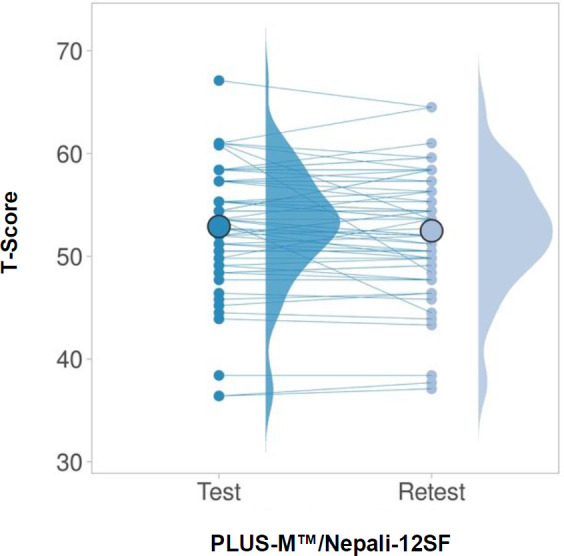
Mean test and retest T-Scores for PLUS-M™/Nepali-12SF

**Figure 4:**
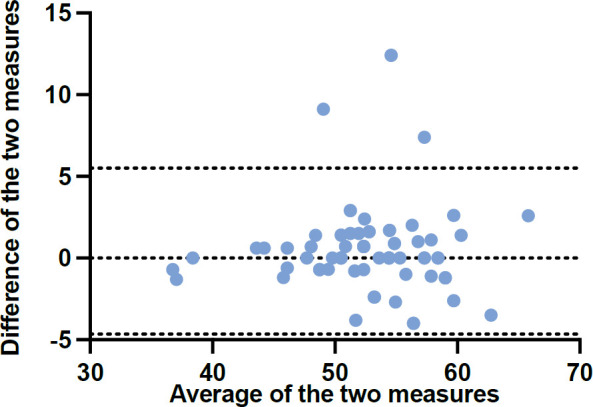
Bland-Altman plot of PLUS-M™/Nepali-12SF. Note: Average of test and retest (x-axis) plotted against difference between test and retest (y-axis). Limits of agreement are represented as dotted lines.

**Figure 5:**
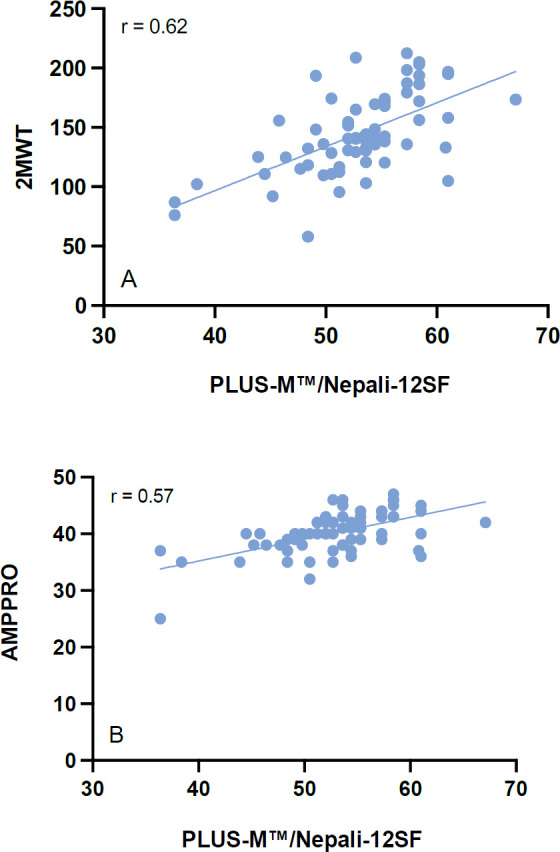
Correlation between PLUS-M™/Nepali-12SF and reference mobility measures. Note: A: PLUS-M™/Nepali-12SF to 2MWT; B: PLUS-M™/Nepali-12SF to AMPPRO.

Good test-retest ICC for T-Score was seen 0.94 (95% confidence interval 0.90–0.96), with initial T-Score being 52.90 ± 5.6 and retest T-Score being 52.47 ± 5.6 (Figure 3 and Figure 4).^18^ Mean distance covered on the 2MWT was 145.45 ± 34.2m and mean AMPPRO score was 40.18 ± 3.8 (K3). Construct validity with the 2MWT was good (r = 0.62, p< 0.001) and moderately positive with the AMPPRO (r = 0.57, p< 0.001) (Figure 5).

## DISCUSSION

The objective of this study was to cross-culturally adapt, translate and evaluate the construct validity of the PLUS-M™/Nepali-12 SF in lower limb prosthesis users residing in Nepal.

Cross-cultural and linguistic translation achieved high internal consistency and T-scores of this study were similar to those seen in the developmental PLUS-M™ study, 52.90 compared to 50.^6^ Our participants still fell within 1 standard deviation of average mobility of over 1,000 lower limb prosthesis users.^19^ Still, our T-Scores were lower than that seen in the French speaking population 56.1.^13^ Regardless, T-Scores indicated that our sample were highly capable of ambulation in their respective environments. Our test-retest findings were excellent and similar to that seen in both the French and original developmental study. Good construct validity was seen with the 2MWT. The 2MWT was an appropriate choice for comparison to the PLUS-M™ as it asks the user to walk at their maximum mobility potential.^20^ Correlation with the AMPPRO was r = 0.57 and compared favorably with that of the original study r = 0.54.

Participant performance in our study evidenced a high mobility and activity level as demonstrated by the AMPPRO, 2MWT and PLUS-M™/Nepali-12SF scores. This may be a result of the younger age, trauma amputation, or trans-tibial level of amputation. As trans-tibial prosthesis users generally have greater mobility and reduced energy expenditure than higher level amputees.^21,22^ PLUS-M™ scores for dysvascular amputees has been reported as 45.3 ± 2.4 which is much lower than our sample but still within 1 standard deviation of mobility of lower limb prosthesis users.

Our participants walked on average 145m during the 2MWT which is much lower than able-bodied persons (183–200m),^23^ but very similar to that of trans-tibial users (147.02 ± 25.9).^24^ Distances covered are a reflection of walking speed, which in our study was 72.5 m/min. Others have observed speeds of 82.3 m/min for trans-tibial and 61.7 m/min for transfemoral users during the 2MWT.^25^ The Amputee Mobility Predictor (AMP) is a popular outcome measure that many clinicians use to categorize amputee activity potential.^26^ K-Level Classification is a commonly used system to classify the functional level of persons with lower limb amputation based on their mobility and ability for potential prosthetic use. The K-Level Classification System indicates a level of functional ability and mobility which commonly ranges from K0-non ambulatory to K4-highly active. In the present study, we saw most of our participants being categorized as K3 level ambulators. Although in the United States, K-level greatly influences prosthetic prescription and resources, it plays little precedent in Nepali health policy.

### Limitations

This study is not without its limitations. Although we recruited a large sample of lower limb amputees, most lost their limbs as a result of trauma which is not a generalization of typical amputation causes. There are reports that nearly 93.4% of amputations are caused by dysvascular reasons.^27^ Moreover, Nepal experienced a major earthquake in the last decade and road traffic trauma is one startling cause of amputation in the region.^28^ Taken together, these two factors may have influenced the sample we relied on for this study. Although it may seem useful to use the PLUS-M™ as a prosthetic outcome measure for new patients, it is recommended to prolong administration until users are accommodated. However, recent scholarship has revealed no worsening or improvement in PLUS-M™ scores from 1 to 7 year.^29^

## CONCLUSION

In this study, we performed cultural contextual translation of a widely used prosthetic mobility outcome measure. Our research evidenced that the PLUS-M™/Nepali-12SF that had excellent reproducibility, meeting standards set forth by the instrument developer for individual comparisons. The significance of this work is that it may allow for the measurement of mobility in austere locations of Nepal. Furthermore, the instrument can be added to performance-based outcome measures to create a broader battery of outcome measures used to understand a prosthesis user's mobility.

The PLUS-M™/Nepali-12SF is currently available for use on the PLUS-M™ website for download and use by clinicians (https://plus-m.org/translations.html). It is hoped that clinicians residing in Nepal can begin to monitor lower limb prosthesis user mobility in an objective, reliable way and simple manner.

## DECLARATION OF CONFLICTING INTERESTS

The authors declare no conflicts of commercial or financial interest in this research.

## AUTHORS CONTRIBUTION

**Amit Ratna Bajracharya:** writing (original drafting)

**Sirirat Seng-iad, Kazuhiko Sasaki, and Gary Guerra:** conceptualization, writing.

All authors have read and agreed to this published version of the manuscript.

## SOURCES OF SUPPORT

This research received no financial support.

## ETHICAL APPROVAL

This study was approved by *Siriraj Institutional Review Board* and also by *Nepal Health Research Council*. All participants provided written informed consent prior to data collection.
